# A New Multi-Axial Functional Stress Analysis Assessing the Longevity of a Ti-6Al-4V Dental Implant Abutment Screw

**DOI:** 10.3390/biomimetics9110689

**Published:** 2024-11-12

**Authors:** Ghada H. Naguib, Ahmed O. Abougazia, Lulwa E. Al-Turki, Hisham A. Mously, Abou Bakr Hossam Hashem, Abdulghani I. Mira, Osama A. Qutub, Abdulelah M. Binmahfooz, Afaf A. Almabadi, Mohamed T. Hamed

**Affiliations:** 1 Department of Restorative Dentistry, Faculty of Dentistry, King Abdulaziz University, P.O. Box 80209, Jeddah 21589, Saudi Arabia; 2 Department of Oral Biology, Cairo University School of Dentistry, Cairo 12613, Egypt; 3 Independent Researcher, Giza 12573, Egypt; ahmedgaziaa@gmail.com; 4 Department of Oral and Maxillofacial Prosthodontics, Faculty of Dentistry, King Abdulaziz University, P.O. Box 80209, Jeddah 21589, Saudi Arabia; lalturki@kau.edu.sa (L.E.A.-T.); hmously@kau.edu.sa (H.A.M.); oqutub@kau.edu.sa (O.A.Q.); abinmahfooz@kau.edu.sa (A.M.B.); aaalmabadi1@kau.edu.sa (A.A.A.); mthamed@kau.edu.sa (M.T.H.); 5 Dental Department, Research Institute of Ophthalmology, Oula, Giza District, Giza 12557, Egypt; aboubakrhashem@yahoo.com; 6 Department of Restorative Dentistry, King Abdulaziz University, P.O. Box 80209, Jeddah 21589, Saudi Arabia; amira@kau.edu.sa; 7 Department of Prosthodontics, Faculty of Dentistry, King Abdulaziz University, P.O. Box 80209, Jeddah 21589, Saudi Arabia

**Keywords:** dental implant therapy, abutment screw, preload, fatigue life prediction, dynamic loading, biomaterials, Ti-6Al-4V alloy, stress distribution, finite element analysis (FEA), fatigue damage parameter, prothesis design

## Abstract

This study investigates the impact of tightening torque (preload) and the friction coefficient on stress generation and fatigue resistance of a Ti-6Al-4V abutment screw with an internal hexagonal connection under dynamic multi-axial masticatory loads in high-cycle fatigue (HCF) conditions. A three-dimensional model of the implant–abutment assembly was simulated using ANSYS Workbench 16.2 computer aided engineering software with chewing forces ranging from 300 N to 1000 N, evaluated over 1.35 × 10^7^ cycles, simulating 15 years of service. Results indicate that the healthy range of normal to maximal mastication forces (300–550 N) preserved the screw’s structural integrity, while higher loads (≥800 N) exceeded the Ti-6Al-4V alloy’s yield strength, indicating a risk of plastic deformation under extreme conditions. Stress peaked near the end of the occluding phase (206.5 ms), marking a critical temporal point for fatigue accumulation. Optimizing the friction coefficient (0.5 µ) and preload management improved stress distribution, minimized fatigue damage, and ensured joint stability. Masticatory forces up to 550 N were well within the abutment screw’s capacity to sustain extended service life and maintain its elastic behavior.

## 1. Introduction

Over the years, continuous advancements in oral implantology and technology have established dental implants as an esthetic and functional definitive restoration for the replacement of missing teeth with a success rate of 90% [1,2,3,4]. Although dental implants are ingrained as an integral part of dental care, several biomechanical complications can occur in clinical practice [5,6,7]. The implant–abutment interface plays a pivotal role in preventing peri-implantitis by minimizing bacterial colonization at this juncture [8,9]. Increasing the contact surfaces at this interface can improve stress distribution, although factors such as crown height, implant connection type, load direction, cusp inclination, occlusal anatomy, and implant position also play significant roles [10]. The stress generated at these interfaces is particularly high in posterior regions, which can affect the abutment’s seating and lead to preload loss under occlusal loading [10,11,12]. Microgaps at the implant–abutment interface can harbor bacteria, facilitating peri-implant tissue contamination [13]. Several factors influence these microgaps, which include vertical misfit between implant components, implant design, applied torque, and screw tightening [13,14]. When planning implant-supported restorations with a two-piece system, attention must be given to the prosthetic screw, a critical area for stress concentration that can lead to biomechanical failure [15]. In implants with internal connections, the abutment screw is a major component that connects the abutment to the osseointegrated implant fixture [16]. The main determinant of the abutment screw stability is the preload, which is the tensile force that clamps the implant–abutment complex parts together using a certain torque [17,18,19]. Optimal preload is necessary to reduce the risk of screw loosening as well as microleakage [20].

The implant assembly is constantly subjected to dynamic loading [21,22,23], where the biomechanical stability of the implant assembly is dependent on the generated magnitude of stress and amount of displacement during function [24,25]. High stresses on the prosthetic parts can lead to the instability of the prosthesis and abutment screw loosening, which will consequently alter the occlusal load distribution and further accelerate the rate of screw loosening [26,27]. This sequence will increase micromotion at the implant–abutment interface and may deteriorate into abutment screw fracture [28,29,30,31]. Accordingly, the most reported mechanical complication is abutment screw loosening or subsequent fracture. This is estimated to have an annual incidence rate of 5.3% in the first year after loading [31,32,33,34], while ranging between 10.4% and 20.8% over a follow-up period of 5 and 10 years, respectively [35].

One of the main factors that contribute to abutment screw loosening is loss of preload. This is evident when the occlusal forces are higher than the achieved preload. Almost 10% of the initial torque application is converted into preload, while the remainder of the torque is utilized to overcome friction due to surface irregularities [36,37,38]. Most manufacturers recommend a tightening torque between 10 and 35 Ncm for optimum preload [39,40]. Additionally, tightening the prosthetic components results in tension followed by compression; however, the percentage of torque to preload is not linear. This may be attributed to several factors, most notably the coefficient of friction as it depends on the tightening speed, surface finish, thread hardness, and presence of lubrication [41].

A study by Hamed et al. [42] recommended against increasing the delivered preload as it may lead to damaging stresses in the implant-complex components and screw overload with deformations. Rather, they advocated scheduling patients for re-tightening to avoid loss of preload and screw loosening. Structural failures in implants can be categorized into immediate fractures from excessive local stress and fatigue-related fractures, the latter being more common [43,44]. Fatigue life experiments, simulating oral conditions, are crucial for understanding the long-term performance of these systems under cyclic loading [45,46]. In this regard, finite element analysis numerical simulations have gained popularity, combining deterministic mechanical behavior with theoretical fatigue analysis. Additionally, the unpredictability of variables in fatigue testing of dental implants can influence the reliability of in vitro experiments [47,48,49,50]. The ISO 14801 standard provides guidelines for high- and low-cycle fatigue simulations of different dental implant designs, materials, and conditions, ensuring consistent comparisons across studies [51]. In the literature, the longevity and mechanical stability of dental implant–abutment assemblies have been extensively explored using finite element models and traditional fatigue criteria, as demonstrated in previous studies [52,53]. However, these studies primarily focused on fatigue life under static or controlled oblique loading conditions, often following ISO 14801 standards, without considering the complex, dynamic nature of chewing forces encountered in clinical settings. Additionally, existing research analyzed fatigue behavior on specific implant components or simplified assemblies, overlooking the cumulative effect of dynamic multi-axial stress on the entire implant prosthetic system. The use of bolt axial tension techniques to simulate preload is also in question, as a direct application and simulation of tightening torque provides more accurate prediction reflective of clinical scenarios [54]. This creates a critical gap in our understanding of how stress exacerbates during different phases of mastication and how prosthetic design elements can be optimized to mitigate failure risks. Accordingly, the objective of this study was to investigate the effect of direct tightening torque application (preload) and the friction coefficient on stress generation and fatigue resistance of a titanium Ti-6Al-4V abutment screw with an internal hexagonal connection under dynamic masticatory loads in high-cycle fatigue (HCF) conditions.

## 2. Materials and Methods

### 2.1. 3D Geometrical Modeling

**Figure 1:**
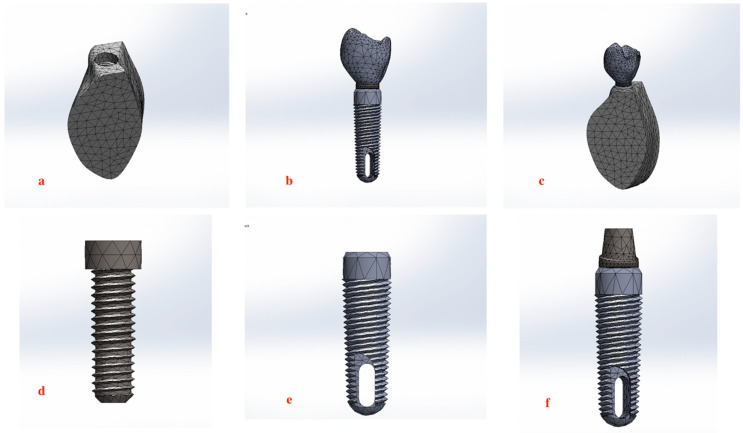
Meshing of structures: (**a**) 3D meshing of bone. (**b**) 3D meshing assembly of implant–abutment and crown complex. (**c**) 3D meshing of final model within bone. (**d**) 3D meshing of abutment screw. (**e**) 3D meshing of implant fixture. (**f**) 3D meshing of implant–abutment assembly.

A three-dimensional (3D) parametric model of a Ti-6Al-4V titanium implant fixture, abutment screw, and an abutment with a screw-retained porcelain crown replacing the mandibular first molar (Ø 5.2 mm, length 12 mm; Implant Direct LLC, Malibu Hills, CA, USA), was constructed using SolidWorks Premium 2010 SP0.0 to ensure precise geometry and alignment (Dassault Systèmes SolidWorks Corporation, Waltham, MA, USA). The implant was modeled to be inserted in the site of a missing mandibular first molar in an adult mandible. The bucco-lingual width of the bone was approximately 11 mm, with the cortical bone surrounding the cancellous bone, having a thickness of about 2 mm. The implant’s axis was aligned with the midpoint of the occlusal table, ensuring ideal placement and inclination relative to the surrounding bone structure [Figure 1a–f].

### 2.2. Implant and Prosthesis Assembly

**Figure 2:**
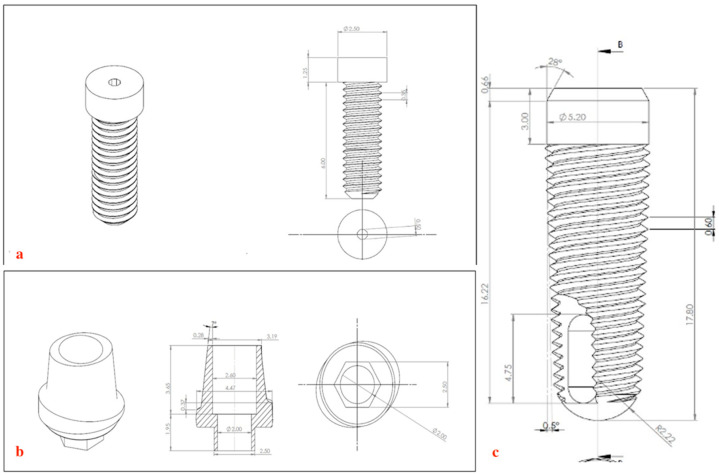
Implant dimensions and geometry: (**a**) Implant abutment screw. (**b**) Implant abutment. (**c**) Implant fixture.

**Figure 3:**
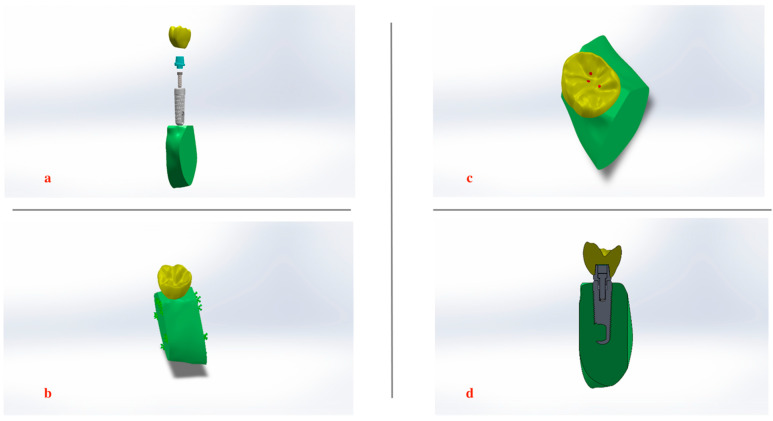
Isometric view of the assembled model: (**a**) All Components of 3D finite element model: bone, implant fixture, abutment, abutment retaining screw, and crown. (**b**) Mandible fixation points. (**c**) Contact areas on occlusal surface of mandibular molar. (**d**) Cross-sectional view of the assembled model components.

A failed ScrewPlant implant system was utilized to obtain precise measurements of the Ti-6Al-4V implant, abutment, and screw dimensions to be modeled according to the manufacturer’s information (Implant Direct LLC, Malibu Hills, CA, USA). The implant was characterized as being spiral (self-tapping) and conical in shape with crystal mini-threads (2–2.5 mm), double lead threads till the implant apex, and an internal hex platform (2 mm long) [Figure 2a–c]. After securing the implant in place, where the axis of the implant coincided with the midpoint of the occlusal table to allow the implant to sit in an ideal position and inclination in relation to the surrounding bone. An integrated abutment screw with internal hexagonal connection was used to secure the abutment and the porcelain crown to the implant fixture, tightened with a recommended torque of 0.3 Nm. The crown featured two buccal cusps, two lingual cusps, and a smaller distobuccal cusp. Properties of all materials are presented in Table 1. Isometric views of the assembled model and its corresponding cross-section are presented in Figure 3a–d.

### 2.3. Finite Element Analysis (FEA)

**Figure 4:**
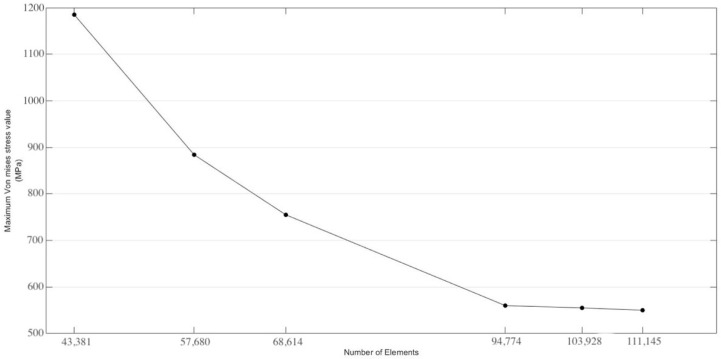
Mesh sensitivity analysis.

The constructed model was exported to ANSYS Workbench 16.2 computer-aided engineering software (ANSYS, Inc., Canonsburg, PA, USA) for mesh generation and finite element analysis (FEA). Within ANSYS, a tetrahedral mesh was applied to the model, with element sizes varying between 0.035 mm and 0.73 mm to capture fine details in the complex geometry of the implant–abutment assembly and ensure precise stress distribution analysis across critical regions (implant fixture, abutment screw, implant–abutment interface). A three-dimensional mesh was generated as shown in Figure 1 and Figure 3 with 180,398 nodes and 103,928 iso-parametric elements, ensuring smooth transitions with a growth rate of 1.2. A convergence analysis was performed to determine the minimum number of elements required for reliable numerical results. Several analyses were conducted with mesh sizes ranging from approximately 43,000 to 111,000 elements, as shown in Figure 4. Initially, the stress values varied significantly and were deemed unreliable. However, once the mesh size exceeded 94,000 elements, the results stabilized and remained consistent with about ±5% change. The materials used in the analysis are biocompatible and were assumed to be isotropic, homogeneous, and linearly elastic [64,65,66].

According to Janeček et al. [67], The S–N curve, or Wöhler curve, graphically represents the number of cycles an implant can endure before fracture under varying cyclic loads. Theoretical modeling allows for the S–N field to serve as an accelerated life testing representation, enabling long-term mechanical response predictions. Utilizing the titanium alloy S–N curve for fatigue prediction is a reliable method for estimating implant survival rates against materials yield strength [68].

### 2.4. Boundary Conditions and Loading

The simulation required careful application of boundary conditions and loading to accurately reflect the physical environment of the dental implant assembly. The inferior surface of the cortical bone was immobilized [Figure 3a], ensuring that no movement could occur at the base of the bone structure. Additionally, mesiodistal displacements along both the mesial and distal planes of the model were restricted, thereby simulating the natural constraints within the mandibular bone.

All contact interfaces between the components of the assembly such as between the implant, abutment interface, and bone were assumed as general contact with a friction coefficient of 0.3 (µ). This means that the different parts of the assembly remained in continuous contact during the simulation, accurately reflecting typical values reported for titanium–titanium interfaces under dry or minimally lubricated conditions, which simulate real-world clinical environments [58,69,70]. This ensures realistic modeling of contact behavior between the abutment screw and implant fixture during mastication. However, the Ti-6Al-4V screw was modeled without accounting for surface coatings, which are often applied via plasma treatment to reduce ion release and minimize toxicological risks. While such coatings improve biocompatibility by preventing the release of vanadium and aluminum ions, they also alter the friction coefficient at the interface. This simplification allowed for an isolated evaluation of the mechanical performance of the implant–abutment complex under multi-axial dynamic loading.

For the simulation of the screw tightening process, A tightening torque of 0.3 Nm was selected based on the manufacturer’s recommendation for internal hexagonal connections. This value is designed to achieve optimal preload, minimizing the risk of screw loosening and micromotion under functional loading while avoiding excessive stress that could lead to plastic deformation or material fatigue. Contact surfaces between the screw, abutment, and implant were critical. These surfaces were designed to transmit specific loads to the screw head during tightening. The loads applied to the screw included the following:

- A tightening torque *M*: This torque was applied to the top surface of the screw, using the recommended value for the tightening moment (0.3 Nm).
- A frictional resisting moment *Mc*: This moment acted on the lower contact surface of the screw, opposing the screw’s rotation.
- An axial force *Fa*: This force was exerted on the lower contact surface of the screw, opposing its advancement during the tightening process.

### 2.5. Screw Tightening and Preload Simulation

**Figure 5:**
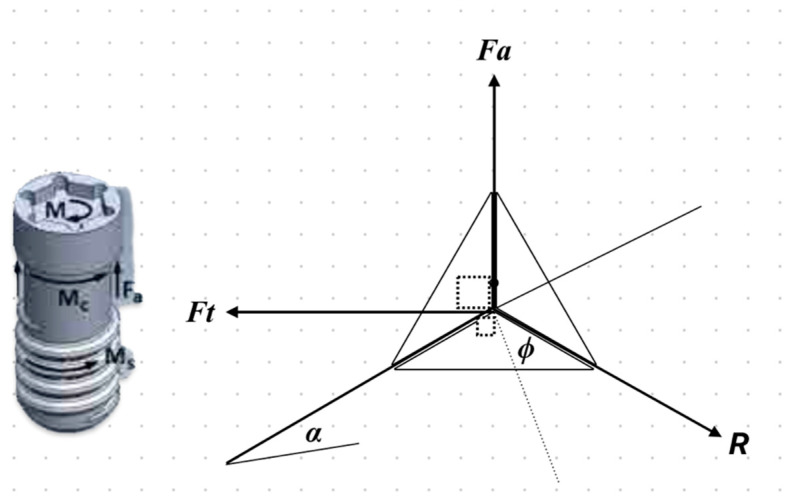
Forces and moments acting on the abutment screw during tightening.

The process of screw tightening was analyzed by considering the moments and forces involved during the advancement of the screw against an opposing axial force (*Fa*) [Figure 5]. The screw’s thread, characterized by a mean radius *r*_mean_ and a helix angle α, interacts with the nut thread surface, where the coefficient of friction μ plays a significant role. During tightening, a turning moment *M*_s_ is applied to the screw’s head to overcome friction on its threaded surface. This is accomplished by generating a tangential force *F_t_*, which attempts to move the screw downward along an inclined surface analogous to the screw thread. The relationship is given by

*M_s_* = *F_t_* · *r*_mean_. (1)

The reaction force *R* from the nut thread surface acts on the screw thread surface, deviating upwards from the direction normal to the inclined surface by the friction angle ϕ, where:

μ = tan(ϕ)
(2)

The forces *F_t_*, *F*_a_, and *R*, are in equilibrium, and the tangential force is given by:

*F*_t_ = *F*_a_ · tan(ϕ + α) (3)

Thus, the moment *M*_s_ required to overcome friction is

*M*_s_ = *F*_a_ · *r*_mean_ · tan(ϕ + α) (4)

The total moment applied to the screw head *M* must exceed *M*_s_ and includes a frictional resisting moment *M*_c_ on the bottom surface of the screw head, which can be expressed as:

*M*_c_ = μ · *F*_a_ · *R*_mean_. (5)

where *R*_mean_ is the mean radius of the contact area between the screw head and the abutment. Therefore, the total moment *M* required during tightening is:

*M* = *M*_s_ + *M*_c_ = *F*_a_ · [*r*_mean_ · tan(ϕ + α) + μ · *R*_mean_] (6)

In the present work, the coefficient of friction (μ) was set to 0.5. With the given screw dimensions, both the axial force (*F*_a_) and the collar friction moment (*M*_c_) were calculated accordingly (specific numerical values to be inserted based on dimensions). A linear elastic stress-deformation analysis was implemented using ANSYS software, which employed an iterative technique to solve for the generated stress and deformation due to screw tightening.

The next loading step involved releasing the wrench moment (*M*) and the friction moment (*M*_c_) to subject the system to the axial force (*F*_a_) only. The resulting residual stresses and deformations were then computed using ANSYS. The force *F*_a_ was applied in the form of a pressure (*P*_a_) on the appropriate surfaces. Once the tightening process was completed and the wrench moment (*M*) was removed, the moments (*M*_s_, *M_c_*), and the tangential force (*F_t_*) no longer existed. However, the screw remained under the influence of the axial force (*F*_a_), maintaining its position within the system.

Subsequently, the superstructure was assembled, and the loading cycle was applied to simulate mastication forces during the chewing process.

### 2.6. Chewing Simulation

The occlusal forces and chewing cycles were simulated for the implant-supported crown. The chewing cycle was divided into three phases: opening, closing, and occluding, with a total cycle duration of approximately 720 ms and an occluding phase of 220 ms at a frequency of 83.33 cycles/minute [71,72,73,74,75]. As per ISO 14801 standards, dental implants are required to withstand a minimum of 5 million loading cycles, with a loading frequency ranging from 2 to 15 Hz [51,75]. In this study, we extended the number of cycles to approximately 1.35 × 10^7^ cycles over 15 years of implant life, as an adult typically undergoes about 9 × 10^5^ cycles/year [76,77]. Therefore, the analysis primarily focused on high-cycle fatigue (HCF) [78].

**Figure 6:**
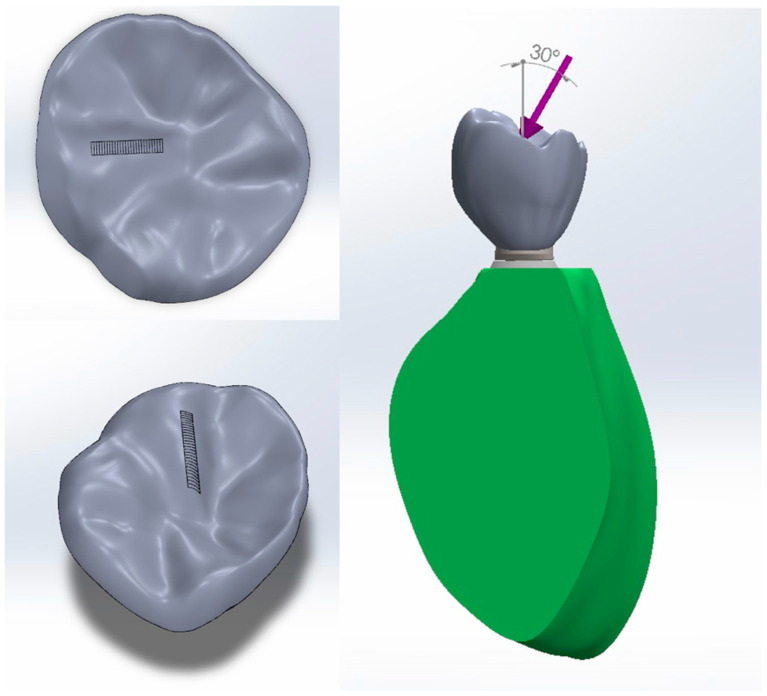
Load application and mastication path.

**Figure 7:**
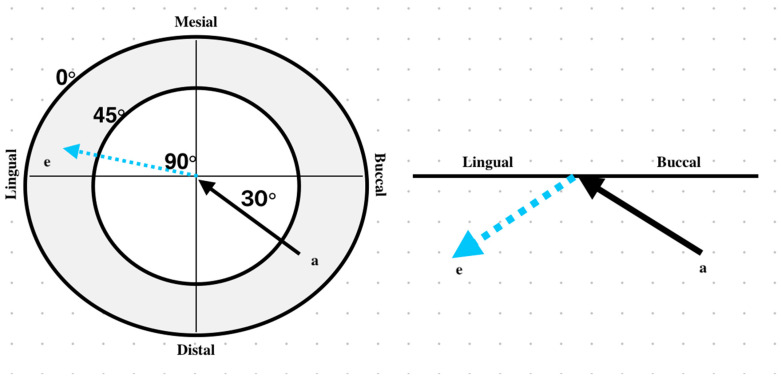
Load angulation and path: point “a” is the start of the traveling path of mastication that ends at point “e”.

**Figure 8:**
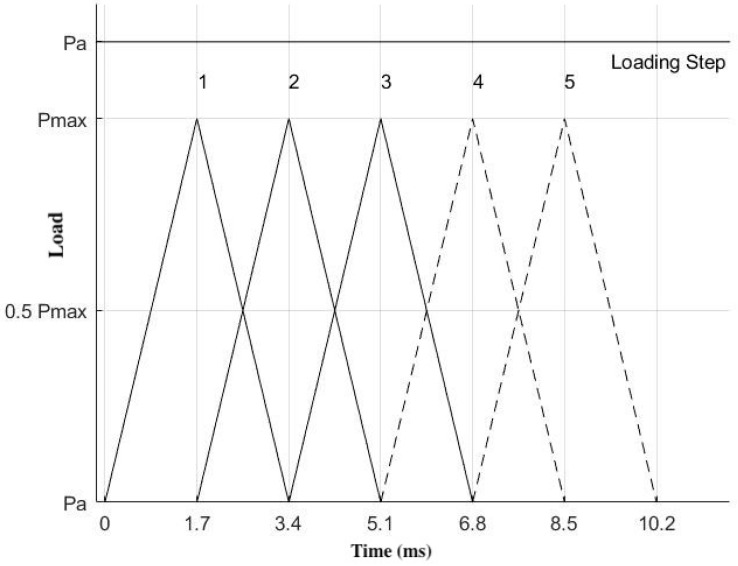
Loading steps invoked in the present idealization. *P*_a_ is the axial pressure applied on the lower surface of the screw head to represent the residual existing due to screw tightening and *P*_max_ is the maximum pressure on the cells of loading during masticatory contact.

During the occluding phase, contact was modeled between the distobuccal cusp of the implant-supported crown and the mesiopalatal cusp of the opposing molar to fulfill normal occlusion. The contact path was represented by a part of the curve resulting from the intersection of the cusp surface with a vertical plane making a contact angle of 30° with the lateral protrusive direction [Figure 6]. The contact started at point ‘a’ and ended at point ‘e’ with a traveling distance of 1.3 mm [Figure 7]. The size of each cell of loading was 4 µm × 10 µm, where loading was applied successively along the contact path. In this study, the maximum pressure P_max_ was assumed to be constant and based on typical chewing forces ranging from 300 N to 1000 N, covering the variability observed in clinical practice. According to the research literature, the average chewing force ranges from 300 to 450 N, with peak forces in healthy individuals reaching 450 to 550 N. In cases of bruxism, these forces can increase to 850 to 920 N [79,80,81]. Although chewing forces fluctuate dynamically, P_max_ was assumed to be constant for simplicity and reflects the peak forces encountered during maximum intercuspation, ensuring a conservative estimate of fatigue performance under high cycle loading conditions [82,83]. The schematic representation of the loading cycles and loading steps is shown in Figure 8.

**Figure 9:**
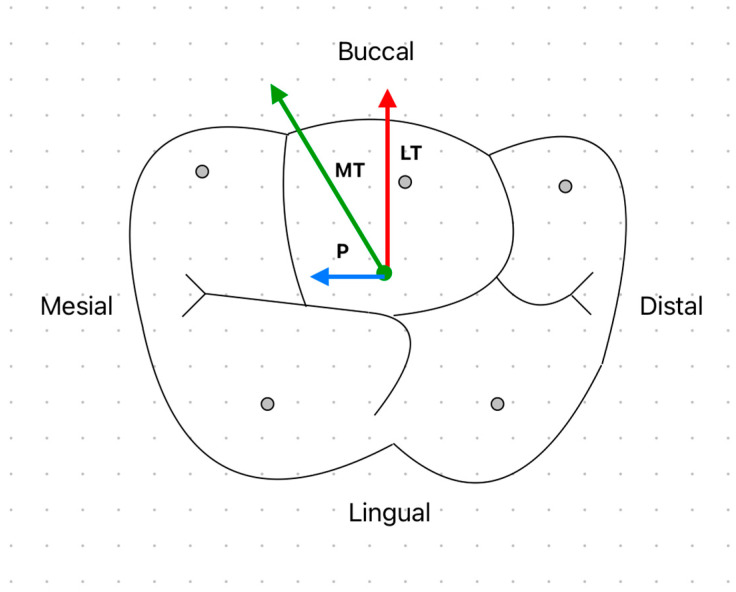
Schematic top view of a first mandible molar to show the limiting borders of possible paths of contact with the opposing maxillary molar during the occluding phase of the chewing cycle. LT: laterotrusive movements, MT: mediotrusive movements, and P: protrusive movements.

The posterior teeth, particularly molars, are naturally positioned to withstand significant masticatory forces, primarily directed along their long axes. Posselt’s envelope of motion provides a comprehensive 3D representation of mandibular movement, analyzing the movement across sagittal, frontal, and horizontal planes [84]. For this study, the horizontal plane is most relevant, particularly for examining cusp paths during occlusal interactions. The “arrow-point tracing” outlines how the functional cusp traces along the mandibular molar’s occlusal surface. Laterotrusive (working side) movement is when the mandible moves laterally (right or left) and the functional cusp moves along the buccal incline of the mandibular molar’s lingual cusps. This movement creates the first arm of the arrow (red), tracing away from the maximum intercuspation. As the mandible moves in the opposite direction, the non-working or mediotrusive movement is created. The maxillary cusp slides along the lingual inclines of the mandibular molar’s buccal cusps. This completes the second arm of the arrow (green). In a forward movement (protrusion), the cusp travels forward on the mandibular molar’s occlusal surface, often moving from the central fossa toward the mesial ridges. This path creates the stem of the arrow (blue), extending anteriorly as the mandible moves forward [85]. The psychological and functional actions of the teeth occur predominantly between the laterotrusive and mediotrusive paths, with most mastication occurring in the lateroprotrusive area. Hence, this study focuses on simulating protrusive, lateroprotrusive, and patrutrusive paths, excluding the non-working path for simplicity [Figure 9]. The multi-axial stress fields generated within the retaining abutment screw under the analyzed loading conditions were evaluated using ANSYS software. The maximum shear stress at each node of the screw was calculated through a two-part process: (a) applying axial preload at the lower surface of the screw head (*σ_o_*) and (b) superimposing an incremental contact load (*P_k_*) during the loading phase.

The difference between the principal stresses *σ_x_* − *σ_y_* at each node was used to define the maximum shear stress range (*τ_xy_*), which was evaluated after each load increment. The node exhibiting the maximum shear stress (*τ_x_*, *τ_y_*) was identified for further analysis. Nodes with *σ_i_* > 0.65*σ_j_* were particularly considered for their significant contribution to stress concentrations and fatigue-related failure mechanisms.

Different magnitudes of mastication forces were applied during the simulation to assess their impact on the fatigue damage experienced by the retaining screw. The forces applied were 300 N, 500 N, 800 N, and 1000 N, representing varying levels of chewing intensity [40,78,86]. These forces were applied to evaluate the fatigue damage parameter experienced by the most stressed site within the retaining screw during the occluding phase of the chewing cycle.

### 2.7. Stress Analysis

The analysis of the multi-axial stress fields generated within the retaining abutment screw due to the loading was carried out as follows. ANSYS provided the maximum shear stress at each node of the screw due to the axial preload σo and the superimposed contact load increment P_k_. The maximum shear stress range τ_xy_ at each node was defined as the absolute difference σ_x_ − σ_y_.

At the end of each loading increment, six independent stresses were computed for each node:

- σ_xx_: Normal stress in the x-direction (axial stress along the x-axis);
- σ_yy_: Normal stress in the y-direction (axial stress along the y-axis);
- σ_zz_: Normal stress in the z-direction (axial stress along the z-axis);
- σ_xy_: Shear stress in the xy-plane (stress acting on the x-face in the y-direction);
- σ_yz_: Shear stress in the yz-plane (stress acting on the y-face in the z-direction);
- σ_zx_: Shear stress in the zx-plane (stress acting on the z-face in the x-direction).

These stresses were used to calculate the Cartesian components of the total shear stress τ_total_ on all possible planes, with angle cosines 30°, 45°, and 65°. The range of τ_x_ and τ_y_ was set from 0 to 1, with an increment of 0.002. The maximum shear stress range Δτ_total_ was then calculated using:

(7) $$∆\tau_{total}=\sqrt{\left(∆{\tau_{total}^{x}}^{2})+(∆{\tau_{total}^{y}}^{2})+(∆{\tau_{total}^{z}}^{2}\right)}$$

This follows the work on Mohr’s Circle [87,88], which is used to determine principal stresses, shear stresses, and normal stresses on any plane in a material under multi-axial loading. Therefore, it helps transform stresses from a general state (combining normal and shear stresses) to principal stresses, which are the maximum or minimum normal stresses on a specific plane with no shear stress. The following damage parameter

**Figure 10:**
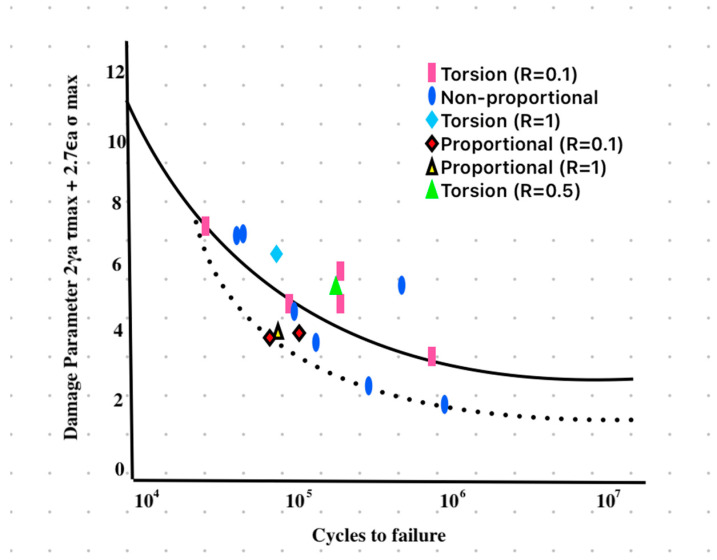
Multi-axial fatigue damage parameter proposed by Kallmeyer et al. [89] to correlate their uniaxial and proportional and non-proportional biaxial experimental fatigue data for Titanium Ti-6Al-4V alloy.

Δτ_total_ = 2γ_a_τ_max_ + 2.7ϵ_a_σ_max_ (8)

was calculated for each node to identify the critical node and its corresponding maximum von Mises equivalent stress σeq value in MPa [89] [Figure 10]. Furthermore, this multi-axial damage parameter was used to estimate the fatigue life of the screw due to the applied contact load. The analysis enabled the estimation of the screw’s endurance under different masticatory forces and the evaluation of its integrity against service mastication forces within the mandibular molar region. The maximum von Mises equivalent stress for each element was calculated for each magnitude of applied mastication force (300 N, 500 N, 800 N, 1000 N) to the implant fixture and abutment retaining screw. To enhance the visualization and interpretation of findings, stress distributions were presented in color gradients, where red signifies zones of highest stress and blue indicates regions of minimal stress, for the clear identification of areas experiencing maximum stress across all models. Each color on the scale was assigned a value in megapascals (MPa).

### 2.8. Fatigue Life Prediction

In this study, fatigue life was assessed using the maximum von Mises equivalent stress ($\sigma_{eq}$) to account for the multi-axial stress conditions present in the abutment screw during functional loading [78,86,89,90,91]. The von Mises stress criterion provides a comprehensive approach for evaluating fatigue under complex loading scenarios, where stresses act in multiple directions simultaneously. According to Jamshidinia et al. [78], von Mises equivalent stress is widely employed in multi-axial fatigue calculations, as it effectively captures the combined effects of normal and shear stresses within the material. This approach is particularly well-suited for materials like Ti-6Al-4V, which experience high-cycle fatigue (HCF) in dental implant systems.

Fatigue analysis is typically conducted using three major methods: strain-life, stress-life, and fracture mechanics. The stress-life (S–N) method, used in this study, is particularly suited for high-cycle fatigue (HCF), where a structure is subjected to millions of cycles under lower cyclic loads. In HCF, almost 95% of the fatigue life is dedicated to the crack initiation stage, making crack prevention a key aspect in improving the fatigue resistance of a structure [92,93]. Furthermore, stress-life traditionally deals with a high number of cycles (greater than 10^5^ cycles), which aligns with the requirements of the ISO 14801 standard for fatigue testing of dental implants [51].

Given that mechanical components rarely experience fully reversed loading conditions, mean stress correction methods were applied to account for the effects of mean stress on fatigue resistance. In this study, the Goodman, Soderberg, and Gerber methods were used to model fatigue behavior under four levels of cyclic loading [78,86,89,90,91,92,93]. The general equation used to calculate the stress amplitude (*σ_a_*) under mean stress is:

(9) $${\sigma_{a}=\sigma_{e}\left(1-\frac{\sigma_{m}}{\sigma_{u}}\right)}^{x}$$

where:

- x=1 for Goodman and Soderberg, and x=2 for Gerber.
- $\sigma_{a}$ is the stress amplitude.
- $\sigma_{e}$ is the fatigue limit.
- $\sigma_{m}$ is the mean stress.
- $\sigma_{u}$ is the ultimate tensile strength.
- In the Soderberg method, $\sigma_{y}$ is used instead of $\sigma_{u}$ for a more conservative fatigue estimate.

By applying these correction methods, the influence of mean stress on the fatigue performance of the abutment screw was carefully modeled, ensuring that the implant system’s fatigue life was evaluated under realistic functional loading conditions. Mean stress corrections, combined with stress-life curves and von Mises equivalent stress, provided an accurate and conservative fatigue life estimation. This comprehensive approach allowed us to predict the behavior of the implant system under simulated oral functional loads, enhancing its fatigue performance in a high-cycle fatigue (HCF) context. Accordingly, the fatigue damage parameter was measured in MPa derived from the maximum von Mises equivalent stress. The graph in Figure 10 illustrates the multi-axial fatigue damage parameter for Ti-6Al-4V, as proposed by Kallmeyer et al. [89], showing the correlation between damage parameters and cycles to failure under different proportional and non-proportional loading conditions. These loading conditions are evaluated for both torsion and combined axial-torsional fatigue experiments. The data points represent various stress ratios (R) with Torsion R = 0.1, Torsion R = −1, Torsion R = 0.5, and proportional and non-proportional tests. The fatigue damage model assumes a mixture of elastic and plastic strain components, especially under high-cycle fatigue conditions, common for Ti-6Al-4V alloy. The use of a fatigue damage parameter for calculating the life expectancy or fatigue life of a titanium implant screw is a valid method and utilizes the maximum von Mises equivalent stress to account for the multi-axial stress conditions that the implant screw experiences in the oral environment. It provides a quantitative approach to assess the gradual accumulation of damage under functional loading conditions. In addition, it allows for a more comprehensive representation of the complex, multi-directional stresses acting on the implant–abutment assembly, which is critical for components like implant screws that experience repeated loads over their lifespan [94,95,96,97].

## 3. Results

### 3.1. Finite Element Analysis of Abutment Screw Stresses and Strains

The finite element analysis (FEA) was performed for each load increment to evaluate the stresses and strains generated at each node of the retaining abutment screw. The loading path was divided into 130 increments, with a constant mastication force applied normal to the mesial surface of the distal cusp. The analysis provided comprehensive data on the stress-deformation fields generated within the different parts of the assembly.

**Figure 11:**
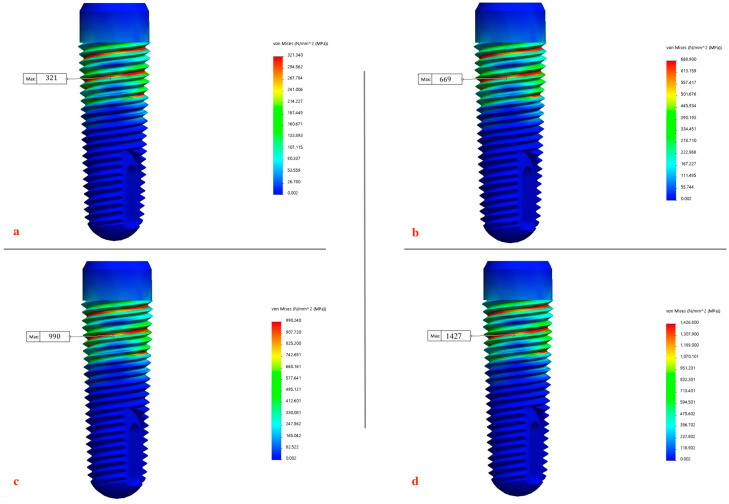
Maximum von Mises equivalent stresses ($\sigma_{eq})$ recorded in implant at different intensities of mastication forces: (**a**) 300 N, (**b**) 500 N, (**c**) 800 N, and (**d**) 1000 N.

**Figure 12:**
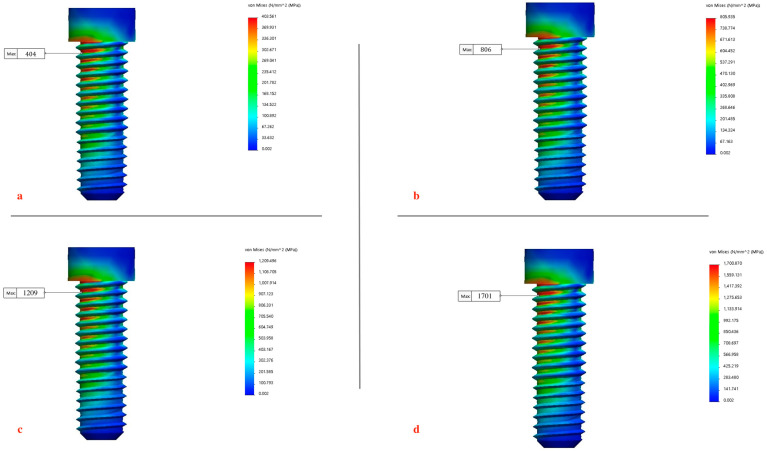
Maximum von Mises equivalent stresses ($\sigma_{eq})$ recorded in abutment screw at different intensities of mastication forces: (**a**) 300 N, (**b**) 500 N, (**c**) 800 N, and (**d**) 1000 N.

The output consisted of six independent stresses, six independent strains, and three Cartesian displacements at each node. The maximum equivalent von Mises stress was selected as a key parameter for comparison due to its relevance to material failure theories under multi-axial stresses. The effects of functional mastication forces on the implant fixture and abutment screw were measured and the maximum von Mises equivalent stresses ($\sigma_{eq})$ were recorded [Table 2] [Figure 11 and Figure 12a–d]:

- At 1000 N: the maximum von Mises equivalent stress recorded a value of 1427 MPa and 1701 Mpa for the implant fixture and abutment screw, respectively.
- At 800 N: the maximum von Mises equivalent stress recorded a value of 990 MPa and 1209 MPa for the implant fixture and abutment screw, respectively.
- At 500 N: the maximum von Mises equivalent stress recorded a value of 669 MPa and 806 MPa for the implant fixture and abutment screw, respectively.
- At 300 N: the maximum von Mises equivalent stress recorded a value of 321 MPa and 404 MPa for the implant fixture and abutment screw, respectively.

### 3.2. Fatigue Damage Analysis During the Chewing Cycle

**Figure 13:**
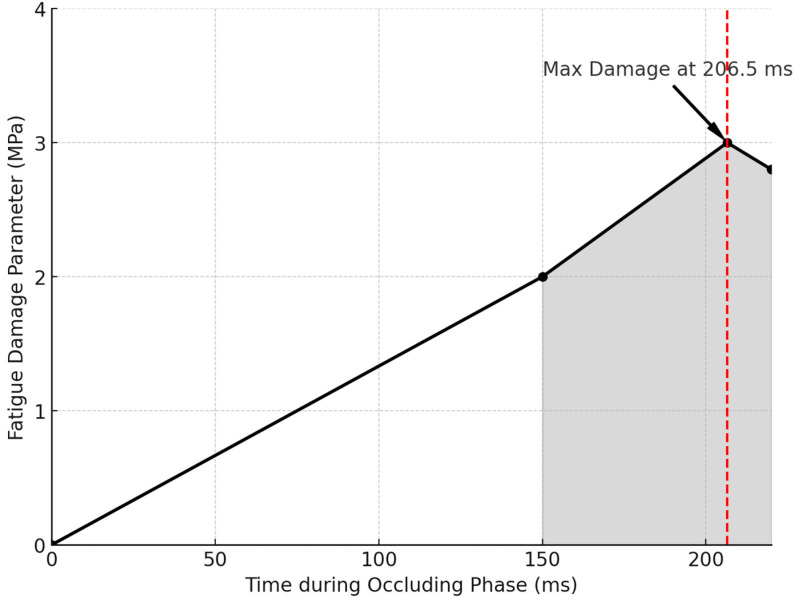
Fatigue damage parameter experienced by the most stressed site within the retaining screw and plotted against contact time during the occluding phase of chewing; maximum fatigue damage invariably takes place at contact time = 206.5 ms.

The fatigue damage parameter, calculated at the most stressed site within the retaining screw, was plotted against contact time during the occluding phase of the chewing cycle under various mastication forces. Figure 13 illustrates that the maximum fatigue damage invariably occurred at contact time—206.5 ms—near the end of the occluding phase.

**Figure 14:**
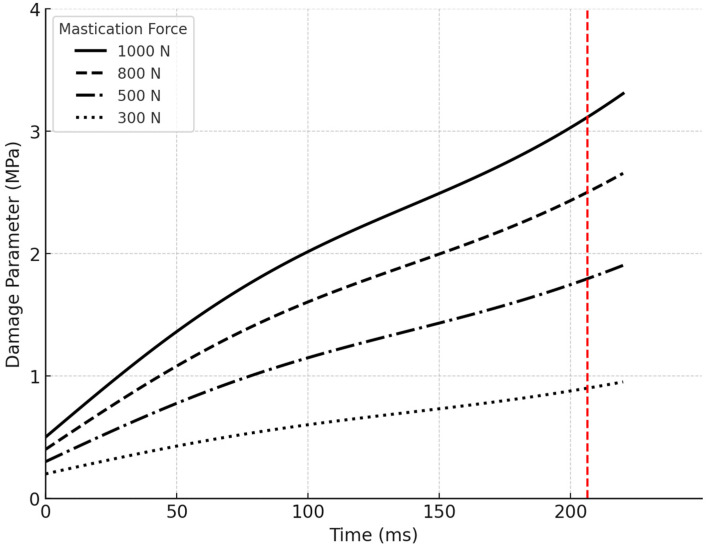
Fatigue damage parameter experienced at contact time = 206.5 by the most stressed site within the retaining screw and plotted against the magnitude of the chewing force.

The impact of different mastication forces on the fatigue damage parameter was meticulously analyzed. As seen in Figure 14, four different chewing forces were considered: 1000 N, 800 N, 500 N, and 300 N. Each force level had a distinct influence on the fatigue damage experienced by the retaining screw.

- At 1000 N: The highest fatigue damage parameter was recorded, reaching a peak value of approximately 3 MPa near the end of the occluding phase at contact time = 206.5 ms. This high-level fatigue damage indicates that the screw is under significant stress, potentially shortening its service life under such intense chewing forces.
- At 800 N: The fatigue damage parameter was slightly lower, peaking at around 2.5 MPa. Although this force is still substantial, the reduction in damage compared to 1000 N suggests that the screw could endure a longer but limited lifespan under these conditions.
- At 500 N: the fatigue damage parameter dropped noticeably to around 1.5 MPa. This lower stress level showed a significant improvement in the screw’s durability, with the potential to withstand a much higher number of cycles without failure.
- At 300 N: The lowest fatigue damage parameter was observed, remaining below 1 MPa throughout the occluding phase. This level of force was well within the screw’s capacity to endure extended service life, likely exceeding the 15-year benchmark typically expected in dental applications.

Furthermore, Figure 14 demonstrates that a fatigue damage parameter below 2 MPa corresponds to an infinite number of cycles, exceeding 1.35 × 10^7^ cycles. In this study chewing forces below 550 N produced a fatigue damage parameter of less than 2 MPa, indicating that the titanium abutment screw could withstand over 1.35 × 10^7^ cycles, equating to at least 15 years of service.

### 3.3. Correlation Between Chewing Force and Fatigue Damage

**Figure 15:**
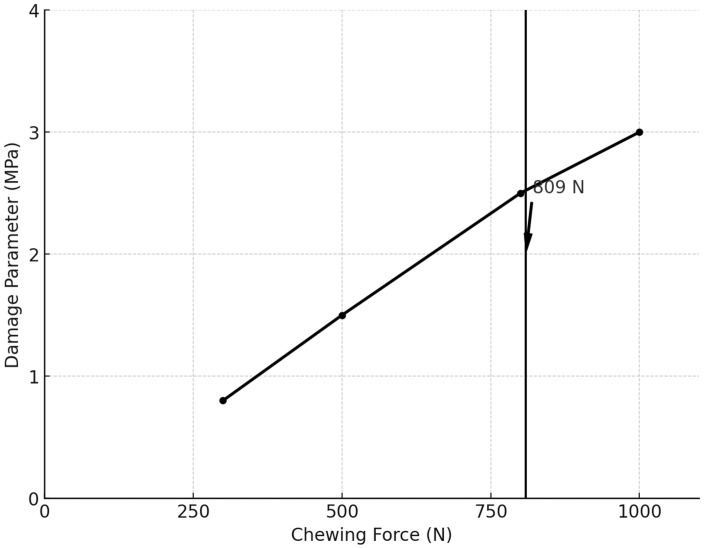
Fatigue damage parameter experienced at contact time = 206.5 ms plotted against the magnitude of the chewing force.

The relationship between chewing force and fatigue damage was further analyzed. Figure 15 presents the fatigue damage parameter experienced at contact time = 206.5 ms plotted against the magnitude of the chewing force. The fatigue damage parameter remained below the Ti-6Al-4V abutment screw material’s yield strength (930 MPa) for chewing forces of 300 N and 500 N, ensuring that the screw could endure infinite cycles. However, for chewing forces of 809 N, the fatigue life of the screw became finite and plastic deformation was indicated.

### 3.4. Effect of Friction Coefficient on Tightening Moments and Axial Pressure

**Figure 16:**
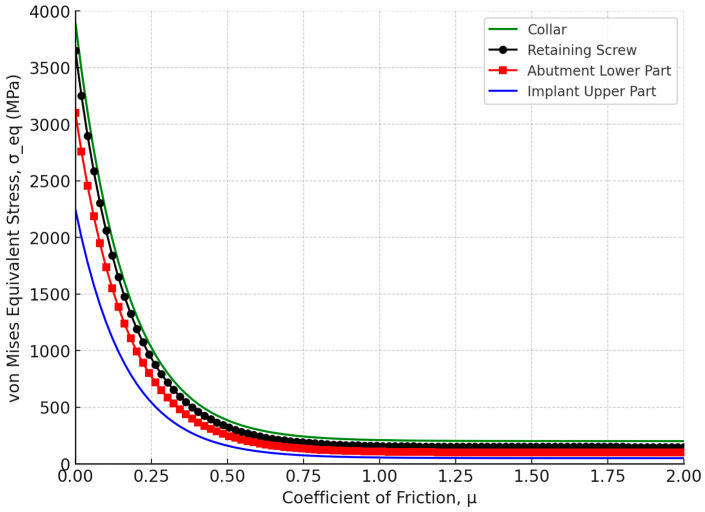
Moment Mc for the screw materials of Ti-6Al-4V titanium alloy versus the coefficient of friction μ.

**Figure 17:**
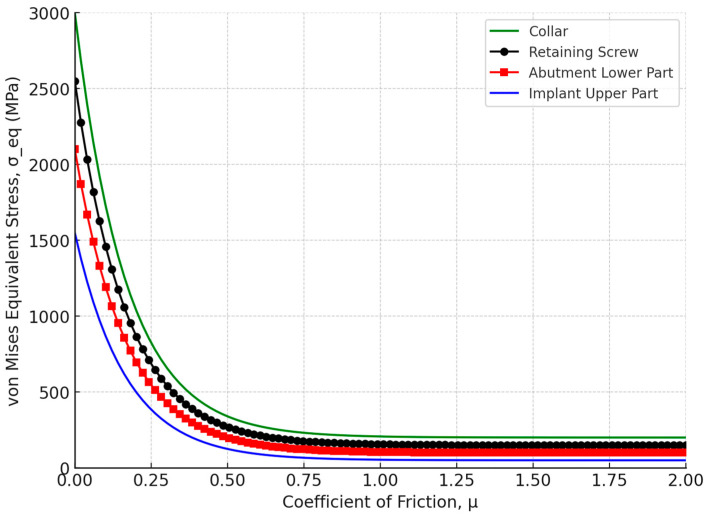
Tightening axial pressure, Pa, plotted against the coefficient of friction μ.

The effect of the coefficient of friction (μ) on the tightening moments and axial pressure (P_a_) for the Ti-6Al-4V titanium alloy was analyzed. The maximum von Mises equivalent stress (σ_eq_) was plotted against the coefficient of friction (μ) for the Ti-6Al-4V titanium alloy retaining screw. The stress values decreased sharply as the coefficient of friction increased from 0 to 0.5 (μ), with a significant reduction in stress levels with higher friction. In Figure 16, the moment M_c_ on the screw material as a function of the coefficient of friction was analyzed. The data reveal that increasing the coefficient of friction from 0 to 0.5 significantly increased the collar friction moment M_c_, while beyond a coefficient of 0.5 (μ), the stress levels stabilized, with the retaining screw exhibiting a maximum equivalent stress of approximately 350 MPa. Moreover, Figure 17 depicts the tightening axial pressure (P_a_) versus the coefficient of friction (μ). Similar to the moment M_c_, the axial pressure exhibited a sharp increase up to a coefficient of friction of 0.5, beyond which the pressure plateaued.

## 4. Discussion

The finite element (FE) method is a widely recognized and effective tool for mechanical analysis. This numerical approach involves breaking down a continuous structure into a finite number of elements for stress and deformation analysis. The FE method excels in handling complex geometries, such as those found in human anatomy, which are difficult to address using traditional analytical methods [98,99,100]. In the field of dental implants, FE analysis has been extensively applied to assess the biomechanical behavior of various prosthetic designs and loading conditions, to examine stress distribution in the supporting bone, and to aid in surgical planning and predict clinical outcomes specific to individual cases [101,102,103,104]. Beyond static stress analysis, FE analysis can also be utilized to estimate the dynamic fatigue lifetime by applying a fatigue post-processor to the stress and strain results under specified loading conditions [105,106]. This study conducted a comprehensive analysis of the longevity of the Ti-6Al-4V titanium abutment screw within a dental implant system, focusing on the impacts of preload, friction, and fatigue life under simulated chewing cycles. The findings highlighted the significant influence of these factors on the mechanical stability and service life of the implant assembly, particularly under varying mastication forces. The correct application of screw preload is paramount to the success of dental implants, as most failures in two-piece dental implants are attributed to screw loosening [107,108,109,110]. This failure often results from micromotion between the implant fixture and the abutment, which leads to a reduction in preload on the connector screw. Conversely, applying excessive preload can damage the screw threads, causing plastic deformation [111,112,113]. Residual stresses induced by high preload levels can exacerbate stress concentration at the screw neck, particularly on the distal surface, leading to accelerated fatigue failure [114]. The use of original abutments is advised to avoid additional tightening and ensure a more precise fit between the implant and abutment, thereby reducing microgap formation and enhancing the longevity of the implant-supported restoration [115,116]. There are two primary methods for applying preload in a finite element model: directly applying the preload torque to the screw [Figure 5] or calculating the bolt axial tension from the recommended torque and applying it to the screw [117,118]. Our results reinforced that simulating preload as direct torque rather than bolt axial tension is optimal, as it creates a secure locking mechanism, which enhances contact pressure under occlusal load and evenly distributes stresses across the implant–abutment complex. Additionally, with the correct amount of preload, the screw can withstand significantly higher loads, reducing the risk of loosening and improving the overall durability and functionality of the implant system, leading to more reliable fatigue resistance under functional masticatory forces. A study by Satpathy et al. [119] reported that preload inclusion via direct torque application in the simulation of clinical dynamic loading increased predicted fatigue lifespan by six orders of magnitude, highlighting that using a simple bolt tension technique for preload simulation resulted in unrealistic stress concentrations. In addition, the authors stated that the loss of preload did not affect the abutment screw’s predicted lifespan until it dropped below 80% of the recommended value (0.3 Nm), underscoring the importance of adhering to manufacturer guidelines. Our results demonstrated that low friction values can lead to stresses higher than the yield stress of the material of some parts of the implant assembly. A sharp decrease in stresses took place as the coefficient of friction values increased from 0 to 0.5 (μ), with the abutment retaining screw showcasing a significant reduction in maximum equivalent stress to approximately 350 MPa [Figure 16 and Figure 17]. This phenomenon can be attributed to the increased resistance to motion at the interface, which stabilizes the mechanical environment of the implant system. Accordingly, the coefficient of friction plays a crucial role in determining the preload delivered during abutment screw tightening. A lower coefficient of friction can be achieved through different methods, for example, lubrication to increase the clamping force, thereby enhancing joint stability [120,121,122]. However, it also introduces a caveat: while reduced friction improves preload, it simultaneously raises stress concentrations on the screw threads, potentially compromising fatigue life, especially under high load conditions [122]. Our findings are consistent with these observations, as the stress levels in our study plateaued at higher friction values, suggesting that there is a critical balance to be struck between friction reduction and stress management.

Our analysis also revealed that increasing the coefficient of friction (*μ*) up to 0.5 resulted in a significant rise in both the collar friction moment *M*_c_ and the tightening axial pressure *P*_a_. Beyond this point, both the moment and pressure plateaued, indicating that further increases in friction have minimal impact [Figure 16 and Figure 17]. This stabilization is crucial as it ensures the screw’s consistent performance under varying friction conditions and highlights the importance of maintaining the coefficient of friction within this range to achieve optimal tightening and uniform stress distribution within the assembly.

**Figure 18:**
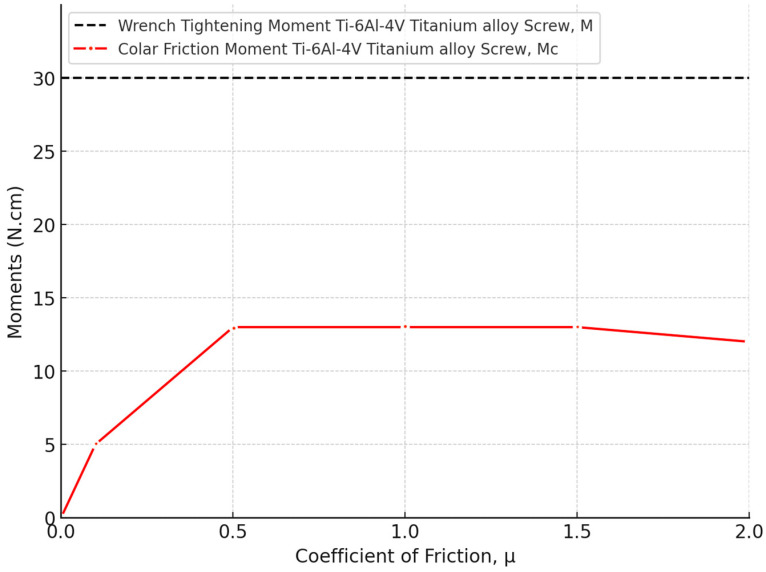
Moment *M*_c_ (frictional resisting moment acting on the lower contact surface of the screw, opposing the screw’s rotation) versus the coefficient of friction μ at a constant tightening torque *M* of 30 N/cm.

Metal fatigue is identified as the primary mechanism of fracture in dental implants [123,124], with the implant–abutment connection often being the weakest point, particularly in internal connection designs that feature a thin fixture wall [125,126]. The magnitude of the average biting force applied to dental implants during mastication is approximately 100 N, but this can increase significantly due to factors such as parafunctional habits like bruxism, the type and location of the implant, and the type of food consumed [69,127,128]. The fracture of implant components is closely linked to stress concentration around the implant system. One of the key factors influencing deformations is increased biting forces during mastication, which can reduce the implant’s fatigue resistance and may lead to mechanical failure of the implant system [128,129,130,131,132]. To evaluate this, the mechanical performance and service life of the Ti-6Al-4V implant model and abutment screw were tested under various loading conditions. The implant was subjected to mastication forces of different intensities 300 N, 350 N, 500 N, 800 N, and 1000 N, with a constant tightening torque of 30 Ncm, and the results are presented in Figure 18, revealing a linear relationship between the applied loads and the resulting stresses. The von Mises stress values obtained from the simulations were compared against the mechanical properties of Ti-6Al-4V alloy used for the implant fixture and abutment screw. Ti-6Al-4V typically exhibits a yield strength of 930 MPa, depending on alloy treatment and processing (ultimate tensile strength (UTS) ranging from 950 MPa to 1200 MPa). The stress results indicate that under the healthy range of normal to maximal mastication forces (300–550 N), the maximum von Mises equivalent stresses remained below the yield strength, ensuring that the components maintain their elastic behavior. Specifically, under normal healthy loads of 300 N load, the stress reached 321 MPa in the implant fixture and 404 MPa in the abutment screw, while in the maximal range of healthy loads of 500 N, the stress increased to 669 MPa in the implant fixture and 806 MPa in the abutment screw [Figure 11 and Figure 12a–d] [Table 2] [79,80,81]. However, at higher loads associated with parafunctional habits or bruxism (800–920 N) [79,80,81], stress levels approached or exceeded the yield strength, indicating a potential for plastic deformation. Specifically, the 800 N load resulted in stresses of 990 MPa in the implant fixture and 1210 MPa in the abutment screw, while the 1000 N load increased the stresses to 1427 MPa and 1701 MPa, respectively. These loads recorded a high damage parameter (2.5–3 MPa) [Figure 14 and Figure 15], suggesting a significant risk of plastic deformation under extreme conditions [Figure 11 and Figure 12a–d] [Table 2]. These findings emphasize the importance of controlling occlusal forces and preload to prevent fatigue failure and ensure the long-term stability of the implant assembly.

These findings align with previous studies that explored the stress behavior of Ti-6Al-4V implants under various loading conditions. Martinez-Mondragon et al. [133] reported von Mises stress values between 745.71 MPa and 786.53 MPa for a 550 N load, using a higher yield strength of 1100 MPa for the Ti-6Al-4V alloy. Their results confirm that stresses near the upper limit of normal occlusal forces still fall below the yield strength in certain implant configurations. Similarly, Abdoli et al. [134] applied loads of 230 N and 270 N at 15 Hz, generating von Mises stress values ranging from 497.4 MPa to 753.8 MPa, which remain within elastic limits. These findings demonstrate that chewing loads at moderate intensities maintain the mechanical integrity of the implant system. In contrast, Bayata et al. [86] explored higher loads, reporting stress values of 845.23 MPa and 931.87 MPa under 500 N and 550 N loads, respectively, against a yield strength value of 930 MPa. These results highlight that masticatory loads exceeding 550 N begin to challenge the material’s mechanical limits, further supporting our findings. Furthermore, Jung et al. [40] demonstrated that even a relatively low load of 100 N generated 563 to 647 MPa in the abutment screw and 849 to 852 MPa in the implant fixture for an internal hexagonal implant system, reflecting the significant stress concentration around the implant–abutment interface even under moderate loading force. This emphasizes the need for regular monitoring and clinical management of implant prosthetics to prevent fatigue-related failures and ensure long-term stability, especially in patients prone to parafunctional activities like bruxism.

In the same manner, the fatigue damage parameter was calculated for the Ti-6Al-4V implant abutment screw based on stress and strain values from experimental tests, which is a common approach for critical plane models [89,93,94,95,96,97]. These models utilize the maximum von Mises equivalent stress to analyze the most damaging stress and strain components on a given plane to predict fatigue crack initiation. The maximum fatigue damage parameter was recorded at the most stressed site within the retaining screw and occurred at contact time = 206.5 ms near the end of the occluding phase of the chewing cycle, particularly under high mastication forces [Figure 13]. Although the guidelines of the Dental Device Branch of the FDA (U.S. Department of Health and Human Services—Food and Drug Administration) recommend a life cycle that is at least more than 5 × 10^6^ cycles (5 million cycles) [135], this study indicated that chewing forces up to 550 N produced a fatigue damage parameter less than 2 MPa, suggesting that the titanium Ti-6Al-4V abutment screw could withstand over 1.35 × 10^7^ cycles (15 million cycles) [Figure 14 and Figure 15]. The fatigue behavior observed in our study aligns with the trends presented by Kallmeyer et al. [89], particularly under higher loads, where non-proportional fatigue behavior reduces the implant’s fatigue life. In Figure 10, the solid line represents proportional loading conditions, where synchronized axial and torsional loads result in more predictable fatigue behavior. In contrast, the dotted line indicates non-proportional loading, where out-of-phase interactions between axial and torsional loads accelerate fatigue damage. Furthermore, our findings offer valuable insights into the temporal dynamics of stresses during complex, multi-directional loading conditions. Thus, it is recommended that the contact duration near the end of the occluding phase of mastication be reduced, as the extension of the occluding path on the implant-supported prosthesis can increase the fatigue damage parameter, potentially shortening the fatigue resistance of the abutment screw in oral service. It also aligns with the general recommendation for implant protheses in the mandibular molar region to exhibit a relatively small occlusal table with conservative anatomical contours.

In clinical practice, what is often observed is the catastrophic failure of a component, which typically takes a much longer time to manifest after the initial crack formation. Fatigue failure generally progresses through three distinct stages: (1) micro-cracks develop in regions with localized stress concentrations; (2) these cracks grow and propagate through the material over time; and (3) the structure experiences sudden and complete failure. The literature suggests that the first stage of crack initiation accounts for up to 90% of the total fatigue life [136]. This aligns with the findings of Shemtov-Yona et al. [137], who reported that 62% of the 100 implants that initially appeared undamaged exhibited cracks or flaws upon spectroscopic analysis, indicating a high likelihood of delayed dynamic failure in the future. Evaluating an implant’s fatigue life may seem sufficient for assessing its durability. However, titanium implants are constantly subjected to dynamic functional loading during oral function, which among other factors, contributes to peri-implant bone resorption. As bone resorption progresses, the bending moments on the implants increase, leading to metal fatigue and potential fracture before the expected lifespan [138]. Thereby, it is recommended that researchers account for the fatigue behavior of implants under conditions of progressive bone resorption, with data that may be derived from clinical trials. 

Prados-Privado et al. [139] conducted an in-depth evaluation of the fracture load and fatigue life of a specific dental implant system using both experimental and numerical analyses via Ansys Software. The study determined that the maximum fracture load was 1000 N, with finite element method (FEM) simulations revealing high stress concentrations at this force. The fracture was consistently located at the first thread of the fixture in both static and cyclic tests, corroborating the FEM results. Wang et al. [125] further investigated the effect of abutment angle on the performance of a two-piece implant system with a taper-integrated screw abutment. Their research, combining numerical modeling with experimental tests, showed that increasing the abutment angle from 6° to 10° resulted in a higher fracture load. Similarly, Raoofi et al. [140] analyzed stress patterns in implant systems with three different connection types: an internal hexagon with a lead-in bevel, an internal connection with a triple channel, and a 110° Morse taper with six anti-rotational grooves. The study found that the second connection type experienced the lowest stress levels due to the larger joint surface, suggesting that abutment design significantly influences the stress distribution and fatigue life of dental implants.

Screw loosening is a prevalent mechanical complication in dental implants, with an incidence rate ranging from 4.3% to 12.7%, as reported in the specialized literature [107,141,142,143]. It has been established that the loosening moment is directly related to the screw preload. Studies demonstrated that the preload value is linearly related to the applied torque moment in butt-joint connections and non-linearly in tapered screw designs [41,144,145,146]. Screw loosening occurs after a specific period of natural use due to cyclic lateral forces that induce rotational movement in the screw, leading to gradual preload loss, a phenomenon known as screw self-loosening [147,148,149,150]. Another reason for screw loosening is the embedment relaxation of mating thread surfaces, which leads to a 10% loss in preload torque. This is particularly evident in newly fabricated rough screws due to a poor machining process, where the applied tightening torque is lost in smoothening the threads rather than settling inside the implant fixture with screw elongation [151,152,153]. Additionally, thread parameters, such as the thread profile’s half angle, significantly influence self-loosening behavior. Nassar et al. [154] found that screws with coarse threads require less loosening torque compared to fine-threaded screws and recommended a higher thread profile angle for enhanced resistance to loosening. Consequently, periodic re-tightening of the abutment screw should be considered to compensate for potential preload loss over time, thereby maintaining implant stability.

The findings from our study highlight the importance of the multifaceted nature of dental implant longevity, where preload management, implant design, and frictional forces all play interconnected roles. Clinicians should ensure that the preload applied to abutment screws is within the manufacturer’s recommended range to optimize the mechanical stability of the implant and reduce the risk of microgaps and screw loosening. Moreover, the study highlights the need for reducing the occlusal table and avoiding full anatomical implant prothesis when placing implants in the mandibular molar area, as this can increase the fatigue damage parameter and reduce the service life of the abutment screw. A recent investigation by Mously et al. [155] explored the influence of occlusal forces on displacement and micromotion in tooth-implant-supported fixed partial dentures. Their findings revealed that even fully osseointegrated implant fixtures exhibit a degree of measurable displacement, which contributes to inevitable micromotion at the implant–abutment interface. The presence of such micromotion is reported as the primary reason for preload loss [41]. This key finding explains the gradual loss of preload under functional masticatory forces and emphasizes the significance of periodic maintenance and screw re-tightening procedures for the long-term stability of implant prothesis. Further future studies examining the effects of displacement and micromotion in relation to screw rotation and loosening are pivotal.

While our study provides valuable insights, it is important to acknowledge its limitations. The finite element analysis (FEA) employed in this study, though comprehensive, may not fully capture the complex interactions present in a clinical environment. The assumptions of linear material properties and idealized boundary conditions might differ from real-world conditions, potentially affecting the generalizability of our findings. Additionally, this study did not account for factors such as embedment relaxation, screw bending, surface manufacturing, or coating variations. The implant–abutment interface was assumed to be ideal, with no irregularities or a misfit between the implant and abutment, where the potential for torque loss over time was not taken into account, simulating a scenario in which the preload remains constant from the beginning of the testing process until fatigue. Moreover, a constant maximum pressure (Pmax) with uniform chewing cycles was assumed, though, in reality, masticatory forces fluctuate with food type, texture, and chewing conditions. Chewing intensities vary between cycles and phases, with peak loads occurring during maximum intercuspation. While this simplification offers a conservative estimate of implant behavior, it may not fully capture real-life variability.

Finally, researchers should consider the impact of peri-implant bone resorption, which increases bending moments on implants over time, leading to premature fatigue failure before the predicted lifetime. Future research should aim to validate these findings via clinical investigations to ensure their applicability across diverse clinical scenarios.

## 5. Conclusions

In this study, the mechanical behavior and fatigue life of a Ti-6Al-4V implant system were analyzed under dynamic mastication forces. Based on the scope and findings of this research, the following conclusions were drawn:

- Applying the manufacturer’s recommended tightening torque (0.3 Nm) ensured mechanical stability and minimized micromotion. Periodic re-tightening is advised to maintain preload over time.
- Direct simulation of tightening torque optimizes stress distribution across the implant–abutment interface, minimizing stress concentrations that could lead to early failure.
- A friction coefficient up to 0.5 (μ) reduced torque-induced stresses, optimizing fatigue resistance without compromising joint stability.
- Masticatory forces up to 550 N maintained the screw’s integrity for over 1.35 × 10^7^ cycles, equivalent to 15 years of implant life. Higher loads (≥800 N) approached or exceeded the Ti-6Al-4V alloy’s yield strength, indicating reduced service life.
- Implant-supported prostheses with smaller occlusal tables and simplified anatomy are recommended for the mandibular molar region to minimize stress accumulation.
- Multi-axial dynamic models offer more accurate fatigue life predictions compared to static models, emphasizing the need to consider full masticatory cycles in implant assessments.

## Figures and Tables

**Table 1:** Material Properties Assigned to the Different Model Components.

Material	Elasticity Modulus	Poisson’s Ratio	Yield Strength
	GPa		
Titanium alloy Ti-6Al-4V	110 [55,56]	0.32 [55,56]	930 [57,58,59]
Cortical bone	13.70 [60,61]	0.30 [60,61]	
Cancellous bone	1.37 [60,61]	0.30 [60,61]	
Porcelain	68.9 [62,63]	0.28 [62,63]	

**Table 2:** The Maximum Von Mises Equivalent Stress Generated in the Titanium Implant Fixture and Ti-6Al-4V Abutment Screw.

	300 N	500 N	800 N	1000 N
Implant	321 MPa	669 MPa	990 MPa	1427 MPa
Abutment Screw	404 MPa	806 MPa	1209 MPa	1701 MPa

## Data Availability

Data are available from the corresponding author upon reasonable request.

## Abbreviations

FEA: Finite Element Analysis, 3D: Three-Dimensional, MPa: Megapascal, Ti-6Al-4V: Titanium alloy (Titanium, 6% Aluminum, 4% Vanadium), μ: Coefficient of Friction, HCF: High-Cycle Fatigue, S–N: Stress-Life Curve, σeq: Equivalent Von Mises Stress, Pa: Axial Pressure, Pmax: Maximum Pressure, LT: Laterotrusive Movements, MT: Mediotrusive Movements, P: Protrusive Movements, ISO: International Organization for Standardization, σa: Stress Amplitude, σe: Fatigue Limit, σm: Mean Stress, σu: Ultimate Tensile Strength, σy: Yield Strength, Mc: Frictional Resisting Moment, Nm: Newton Meter (unit of torque), N: Newton (unit of force), ms: Milliseconds, Fa: Axial Force, rmean: Mean Radius, α: Helix Angle, Ms: Turning Moment, Ft: Tangential Force, R: Reaction Force, ϕ: Friction Angle, M: Total Moment, Rmean: Mean Radius of the Contact Area.
